# Description of *Rhodnius marabaensis* sp. n. (Hemiptera, Reduviidae, Triatominae) from Pará State, Brazil

**DOI:** 10.3897/zookeys.621.9662

**Published:** 2016-10-03

**Authors:** Eder dos Santos Souza, Noé Carlos Barbosa Von Atzingen, Maria Betânia Furtado, Jader de Oliveira, Juliana Damieli Nascimento, Daniel Pagotto Vendrami, Sueli Gardim, João Aristeu da Rosa

**Affiliations:** 1 Faculdade de Ciências Farmacêuticas, Universidade Estadual Paulista “Júlio de Mesquita Filho” (UNESP), Araraquara, SP, Brasil; 2 Fundação Casa da Cultura de Marabá, PA, Brasil FCCM; 3 Instituto de Biologia, Universidade Estadual de Campinas (UNICAMP), SP, Brasil; 4 Instituto de Medicina Tropical de São Paulo – USP, SP, Brasil; 5 Faculdade de Odontologia, Universidade Estadual Paulista “Júlio de Mesquita Filho” (UNESP), Araraquara, SP, Brasil

**Keywords:** Triatominae, Rhodnius marabaensis sp. n., new species, Amazon

## Abstract

*Rhodnius marabaensis* **sp. n.** was collected on 12 May 2014 in the Murumurú Environmental Reserve in the city of Marabá, Pará State, Brazil. This study was based on previous consultation of morphological descriptions of 19 *Rhodnius* species and compared to the identification key for the genus *Rhodnius*. The examination included specimens from 18 *Rhodnius* species held in the Brazilian National and International Triatomine Taxonomy Reference Laboratory in the Oswaldo Cruz Institute in Rio de Janeiro, Brazil. The morphological characteristics of the head, thorax, abdomen, genitalia, and eggs have been determined. *Rhodnius prolixus* and *Rhodnius robustus* were examined in more detail because the BLAST analysis of a cyt-b sequence shows they are closely related to the new species, which also occurs in the northern region of Brazil. The most notable morphological features that distinguish *Rhodnius marabaensis* **sp. n.** are the keel-shaped apex of the head, the length of the second segment of the antennae, the shapes of the prosternum, mesosternum and metasternum, the set of spots on the abdomen, the male genitalia, the posterior and ventral surfaces of the external female genitalia, and the morphological characteristics of the eggs. *Rhodnius jacundaensis* Serra, Serra & Von Atzingen (1980) *nomen nudum* specimens deposited at the Maraba Cultural Center Foundation - MCCF were examined and considered as a synonym of *Rhodnius marabaensis* **sp. n.**

Maraba Cultural Center Foundation -

## Introduction

Vectors of the protozoan *Trypanosoma cruzi*, the etiological agent of Chagas disease, include 151 species distributed into 18 genera belonging to the subfamily Triatominae (Galvão 2014, Mendonça et al. 2016). The genus *Rhodnius* includes 19 species (Alevi et al. 2015), of which six were described after the publication of the Lent and Wygodzinsky review (1979): *Rhodnius stali* Lent, Jurberg & Galvão, 1993; *Rhodnius colombiensis* Mejia, Galvão & Jurberg, 1999; *Rhodnius milesi* (Carcavallo, Rocha, Galvão & Jurberg, 2001); *Rhodnius zeledoni* Jurberg, Rocha & Galvão, 2009; *Rhodnius montenegrensis* Rosa et al., 2012, and *Rhodnius barretti* Abad-Franch, Palomeque & Monteiro, 2013. *Rhodnius amazonicus* Almeida, Santos & Sposina, 1973 was synonymized with *Rhodnius pictipes* by Lent and Wygodzinsky (1979) according a photograph of the holotype, but subsequently it was validated by Bérenger and Pluot-Sigwalt (2002) by morphological study of 19 characters with of the two species.

Among the *Rhodnius* species, only nine are found in the northern region of Brazil: *Rhodnius amazonicus*, *Rhodnius brethesi*, *Rhodnius milesi*, *Rhodnius montenegrensis*, *Rhodnius neglectus*, *Rhodnius paraensis*, *Rhodnius pictipes*, *Rhodnius robustus*, and *Rhodnius stali* (Galvão 2014, Meneguetti et al. 2015).

In May 2014, two *Rhodnius* spp. specimens were collected in Marabá, Pará, Brazil, and compared to the key described by Lent and Wygodzinsky (1979), as well as to previously described *Rhodnius* species, without success. These samples were compared and identified as the same species as *Rhodnius jacundaensis* Serra, Serra & Von Atzingen, 1980 which had been deposited at the Marabá Cultural Center in Pará. *Rhodnius jacundaensis* was mentioned in an abstract presented at the Fourth Annual Brazilian Parasitology Conference in Rio de Janeiro in 1980. According to Article 9 of the International Code of Zoological Nomenclature, however, this new species was not confirmed. As a result, Carcavallo et al. (1998) and Galvão et al. (2003) considered it to be *Rhodnius jacundaensis* Serra, Serra & Von Atzingen, 1980) *nomen nudum*. This article describes *Rhodnius marabaensis* sp. n. as the tenth species found in northern Brazil and the twentieth member of this genus (Abad Franch et al. 2013, Galvão 2014, Meneguetti et al. 2014, Alevi et al. 2015).

## Materials and methods

### Morphological identification and description

**Figure 1.**
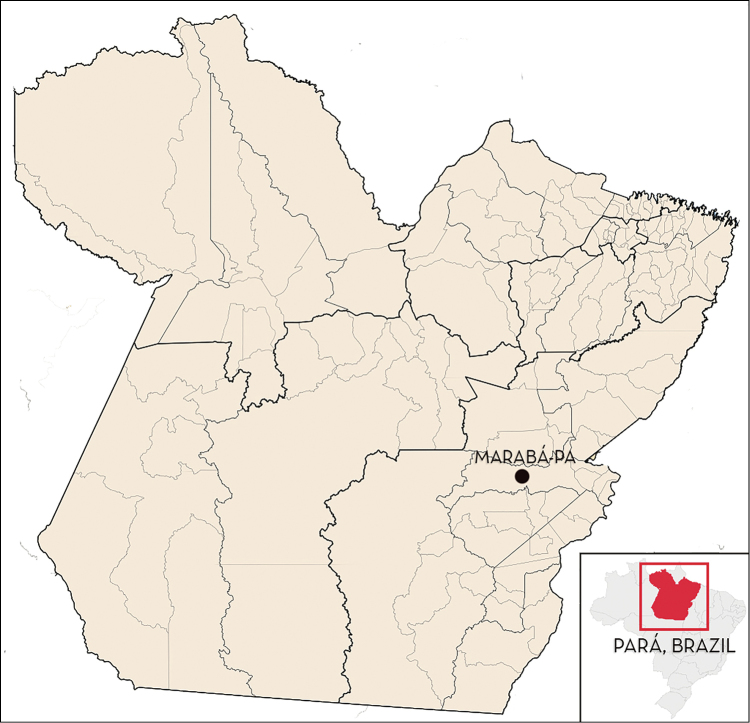
Localization of Marabá- PA where *Rhodnius marabaensis* sp. n. specimens were collected (05°21'54"S, 49°07'24"W).

The specific description was based on the observation of two adult specimens (one female and one male) collected in a residence in the Murumuru Environmental Reserve in the city of Marabá, Pará, Brazil (coordinates: 10°10'05.1"S and 63°24'09.1"W) (Fig. 1). The description included 14 males and 14 females deposited at the MCCF and previously characterized as *Rhodnius jacundaensis* [*nomen nudum*].

The identification of samples was performed using the dichotomous key by Lent and Wygodzinsky (1979). The study also considered descriptions of *Rhodnius amazonicus*, *Rhodnius stali*, *Rhodnius colombiensis*, *Rhodnius milesi*, *Rhodnius zeledoni*, *Rhodnius montenegrensis*, and *Rhodnius barretti* (Almeida et al. 1973, Lent et al. 1999, Mejia et al. 1999, Valente et al. 2001, Jurberg et al. 2009, Rosa et al. 2012, Abad-Franch et al. 2013). *Rhodnius marabaensis* was also compared to specimens of 18 *Rhodnius* species held at the Brazilian National and International Triatomine Taxonomy Reference Laboratory at the Oswaldo Cruz Institute in Rio de Janeiro, Brazil. The only species that was compared only by description was *Rhodnius amazonicus*, which is held at the Brazilian National Institute of Amazon Research (INPA).

### Genetic identification

After the identification, the mitochondrial gene fragment cytochrome b (cyt-b) was amplified by using suggested primers by Monteiro et al. (2003). The amplified fragments were purified and sequenced in duplicate (forward and reverse). The same haplotype was shown with 693 base pairs (bp). This sequence was evaluated by BLAST (http://www.blast.ncbi.nlm.nih.gov/Blast.cgi) to diagnose the homologous sequences in GenBank. In view of this and of the fact that all three species occur in Northern Brazil (Galvão et al. 2003, Galvão 2014), *Rhodnius robustus* and *Rhodnius prolixus* were morphologically examined and compared in more detail.

### Morphological study

**Figure 2.**
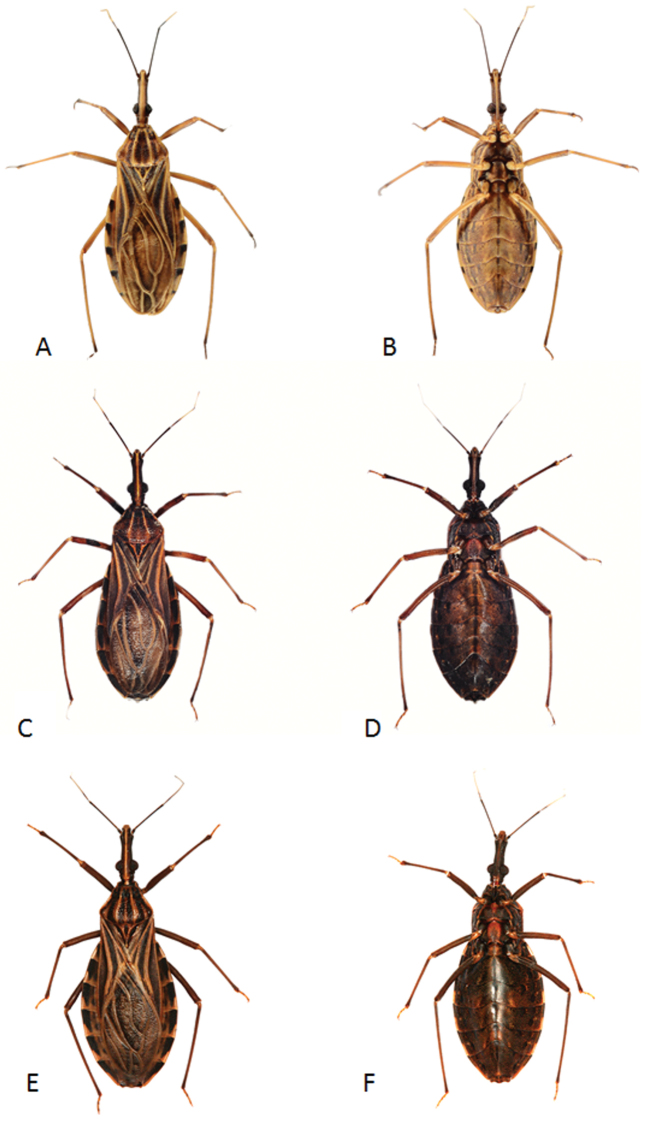
*Rhodnius marabaensis* sp. n. female (**A** dorsal side **B** ventral side); *Rhodnius prolixus* female (**C** dorsal side **D** ventral side); *Rhodnius robustus* female (**E** dorsal side **F** ventral side).

For the comparative morphological study, *Rhodnius prolixus* specimens from Araraquara Triatominae Colony (CTA) 074 were used, as were *Rhodnius robustus* specimens from CTA085. The specimens were kept in these colonies at the Triatominae Insectarium at the School of Pharmaceutical Sciences of São Paulo State University (UNESP), Araraquara, São Paulo, Brazil. The *Rhodnius prolixus* colony had been originally collected in a sylvatic environment in Venezuela on 23 May 1983. The *Rhodnius robustus* colony has been maintained since February 1972 using specimens from Peru (Fig. 2).

**Figure 3.**
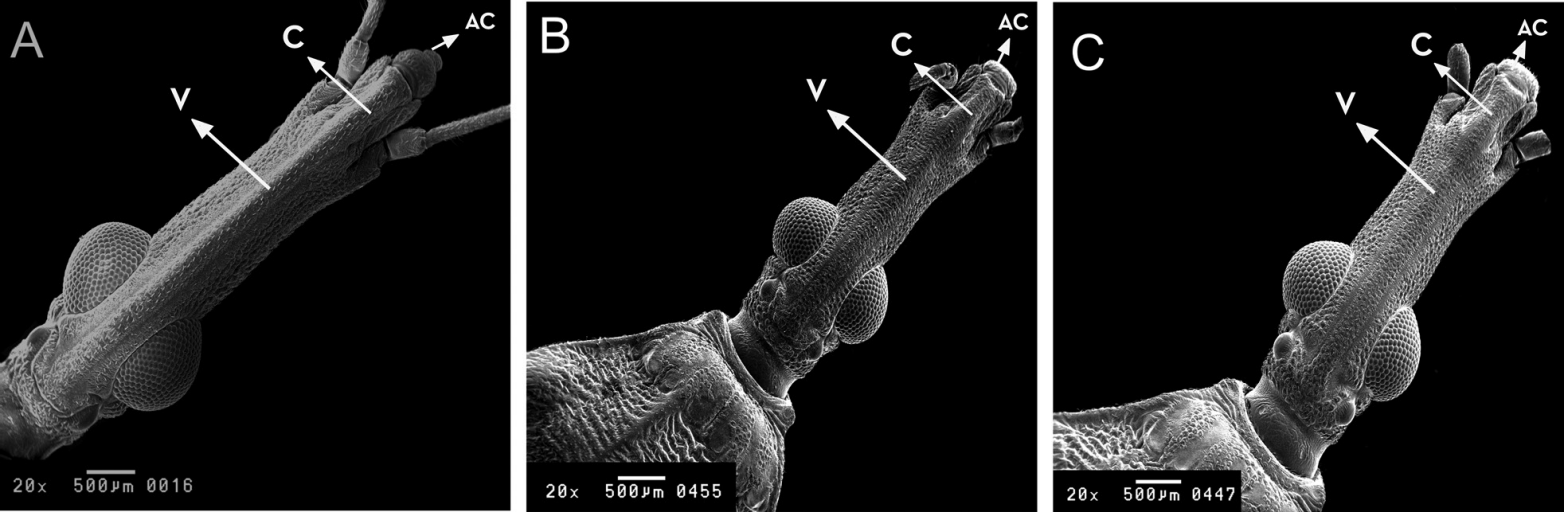
Head of *Rhodnius marabaensis* sp. n. (**A**), *Rhodnius prolixus* (**B**), *Rhodnius robustus* (**C**). V: vertice; C: clypeus; AC: anteclypeus.

**Figure 4.**
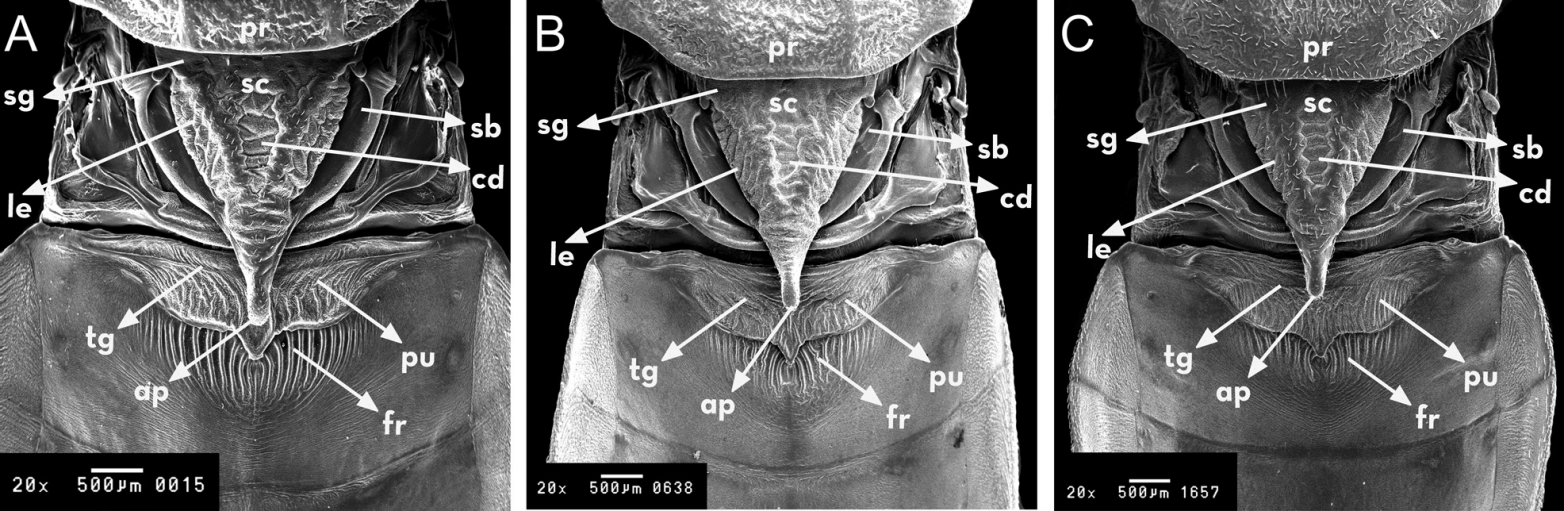
Escutellum and process of I urotergit of *Rhodnius marabaensis* sp. n. (**A**), *Rhodnius prolixus* (**B**), *Rhodnius robustus* (**C**). pr: pronotum; sc: escutelum; sb: semi- circular base; sg: glabrous space; cd: central depression; le: lateral edge; ap: apex of escutelum; pu: process of I urotergit; tg: transverse groove; fr: fringe.

**Figure 5.**
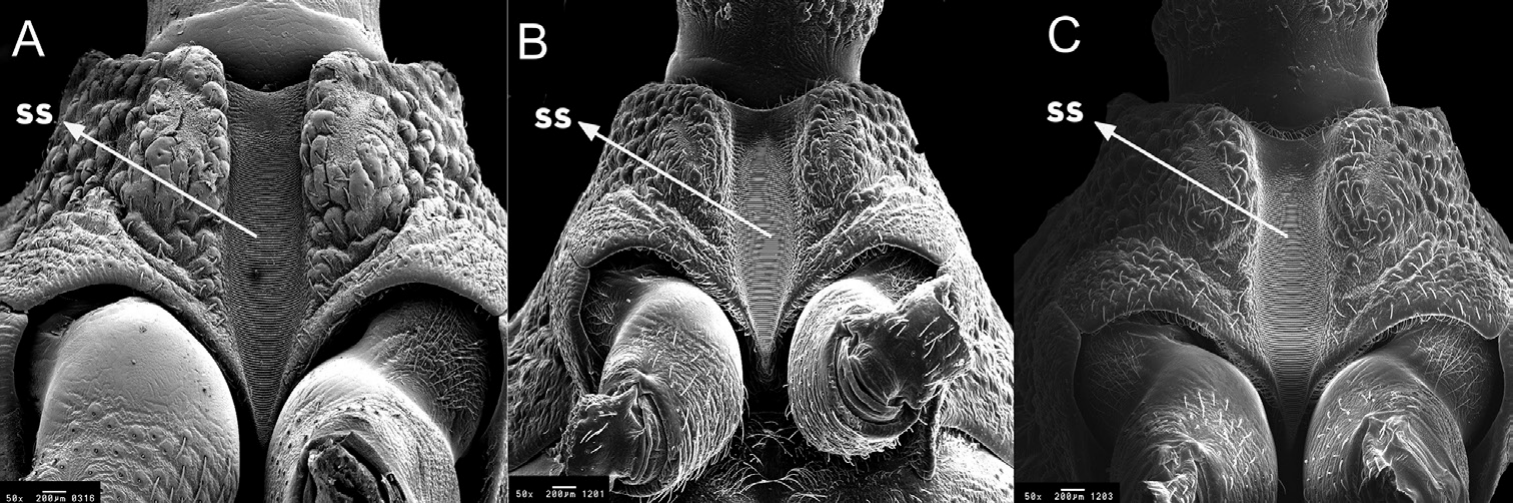
Thorax ventral of *Rhodnius marabaensis* sp. n. (**A**), *Rhodnius prolixus* (**B**), *Rhodnius robustus* (**C**). SS: Stridulatory sulcus.

**Figure 6.**
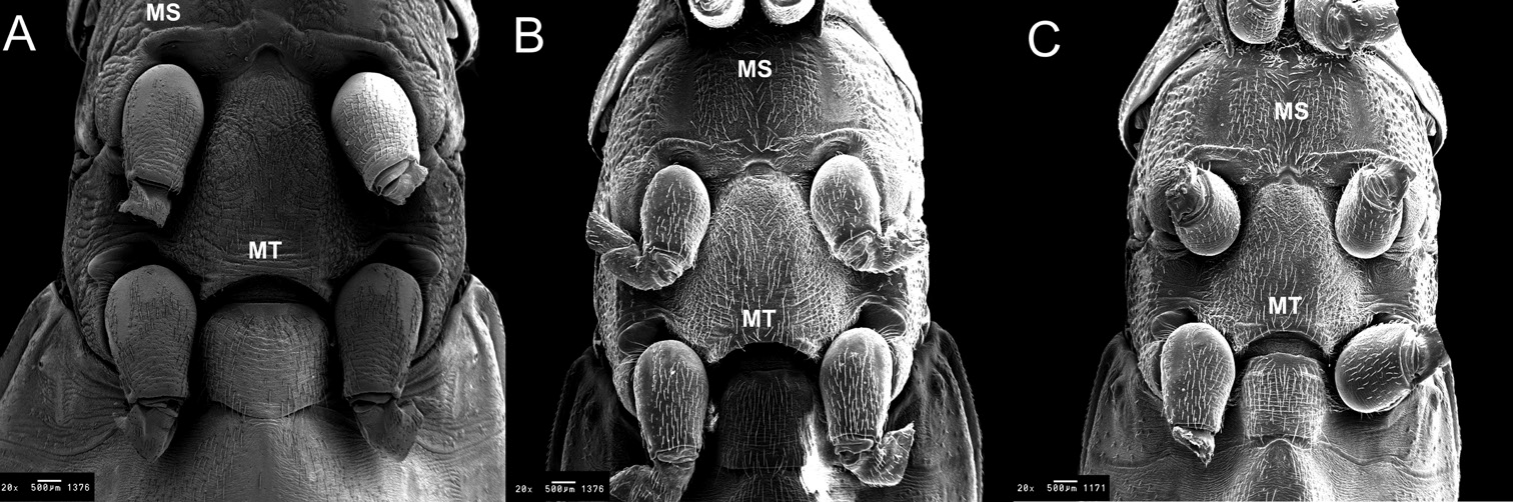
Thorax ventral of *Rhodnius marabaensis* sp. n. (**A**), *Rhodnius prolixus* (**B**), *Rhodnius robustus* (**C**). ms: mesosternum; mt: metasternum.

Optical microscopy and scanning electron microscopy (SEM) were used to compare the morphology of *Rhodnius marabaensis* sp. n., *Rhodnius prolixus*, and *Rhodnius robustus*. The head, the ventral portion of the thorax, the scutellum, and the pygophore were studied using SEM (Figs 3–6, 8). A female of *Rhodnius marabaensis* sp. n. that had been collected in May 2014 was used to study the female genitalia, and 13 eggs obtained from its uterus were also analyzed by SEM (Figs 9–13).

**Figure 7.**
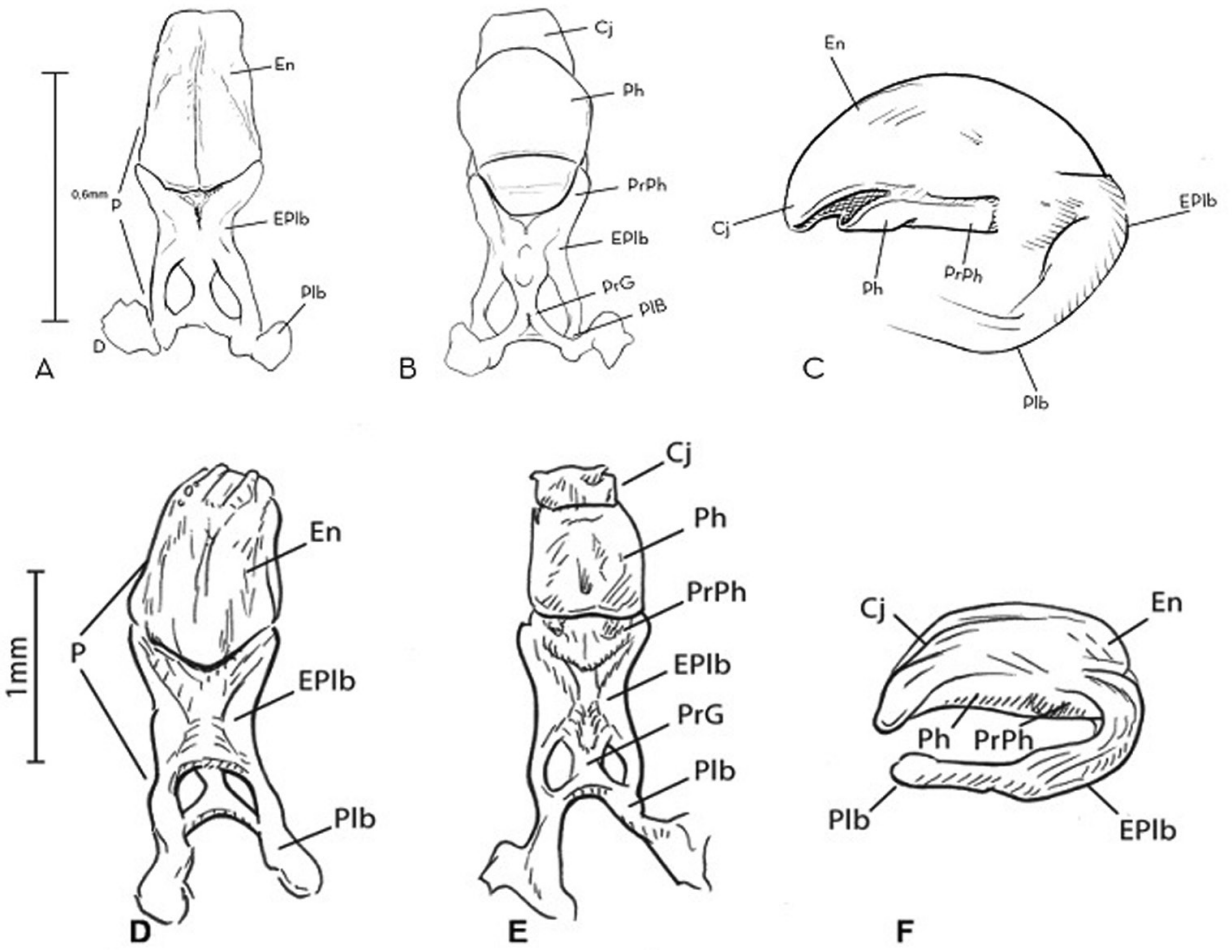
Phallus of *Rhodnius marabaensis* sp. n. (**A** dorsal view **B** ventral view **C** lateral view) and *Rhodnius robustus* (**D** dorsal view **E** ventral view **F** lateral view). Cj: conjunctive; En: endosome; EPlb: median extencion of basal plate; P: phallus; Plb: basal plate; PrG: gonopore process; PrPh: phallossoma process; Ph: phallosoma.

**Figure 8.**
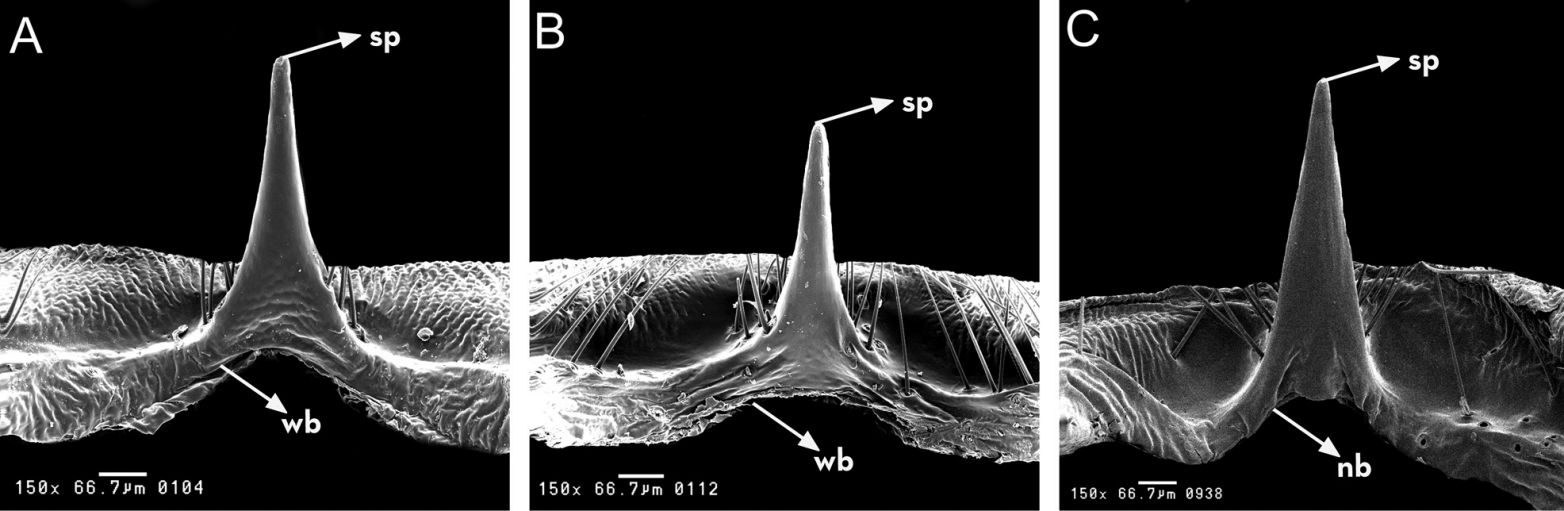
Median process of the pygophore of *Rhodnius marabaensis* sp. n. (**A**), *Rhodnius prolixus* (**B**), *Rhodnius robustus* (**C**). gp: gross point: nb: narrow triangular base; sp: slender point; wb: wide triangular base.

**Figure 9.**
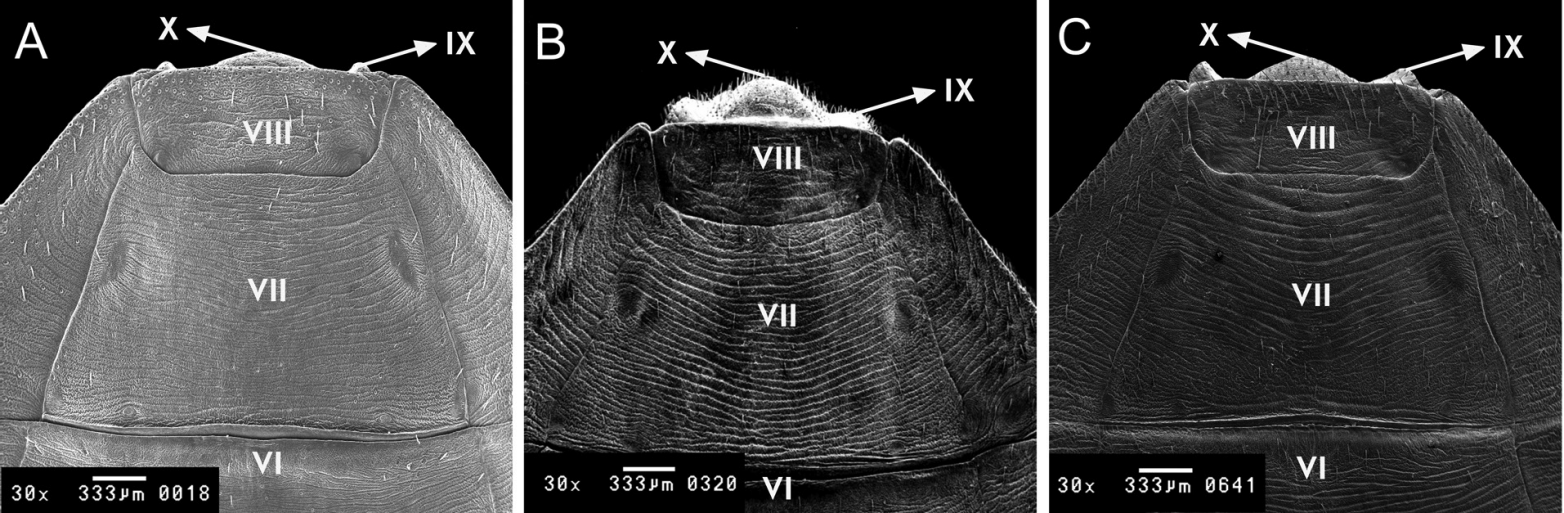
Female external genitalia, dorsal side of *Rhodnius marabaensis* sp. n. (**A**), *Rhodnius prolixus* (**B**), *Rhodnius robustus* (**C**). VI, VII, VIII, IX, X, tergites.

**Figure 10.**
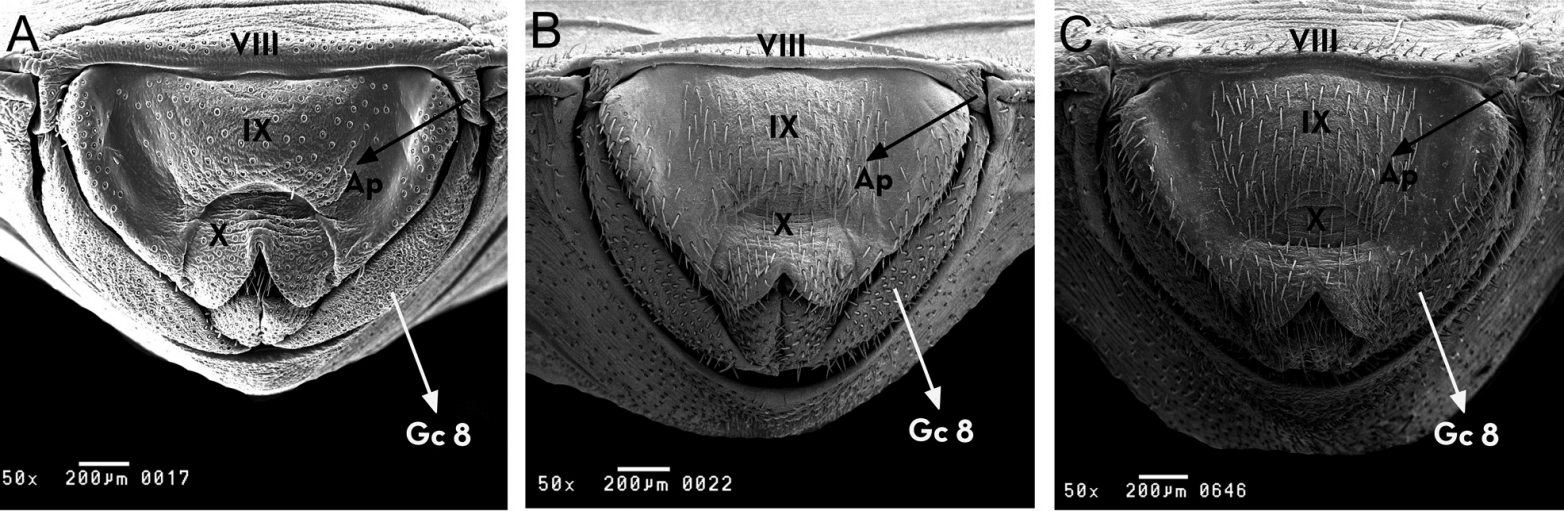
Female external genitalia, posterior side of *Rhodnius marabaensis* sp. n. (**A**), *Rhodnius prolixus* (**B**), *Rhodnius robustus* (**C**). Ap: appendices; Gc 8: gonocoxite VIII; Gp 8: gonaphofyse VIII; VII, VIII, IX: tergites; X: segment.

**Figure 11.**
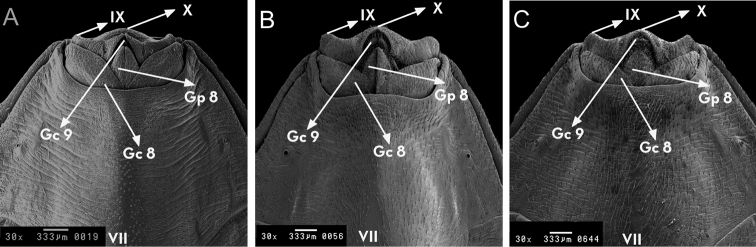
Female external genitalia, ventral side of *Rhodnius marabaensis* sp. n. (**A**), *Rhodnius prolixus* (**B**), *Rhodnius robustus* (**C**). Gc 8: gonocoxite VIII; Gc 9: gonocoxite IX; Gp 8: gonapofhyse VIII; VII, IX: esternites; X: segment.

**Figure 12.**
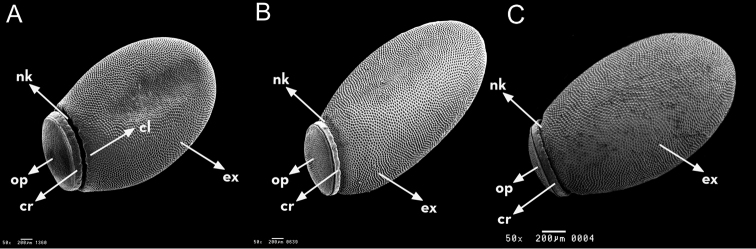
Eggs general vision of *Rhodnius marabaensis* sp. n. (**A**), *Rhodnius prolixus* (**B**), *Rhodnius robustus* (**C**). cl: colar; cr: chorial rim; ex: exochorium; nk: neck; op: operculum.

**Figure 13.**
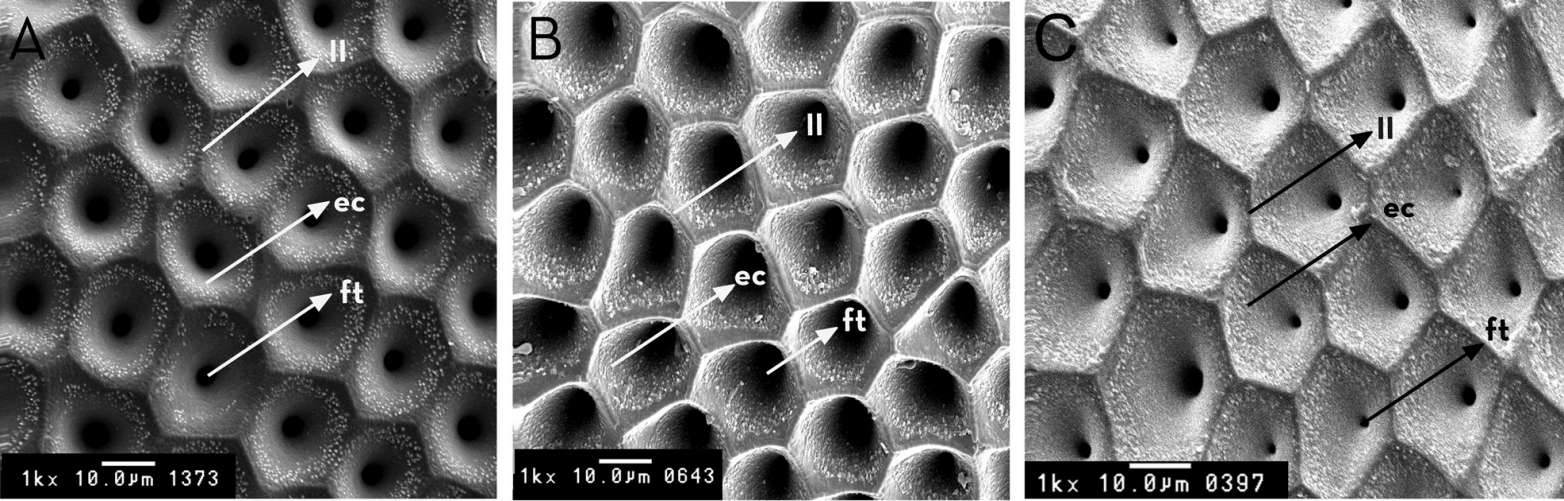
Egg exochorium detail of *Rhodnius marabaensis* sp. n. (**A**), *Rhodnius prolixus* (**B**), *Rhodnius robustus* (**C**). ec: exochorium cell; ft: follicular tubes; ll: limiting line.

### Morphometric study

A Leica MZ APO stereoscope and the Motic Images Advanced System version 3.2 were used for the measurements, as well as for the study of *Rhodnius marabaensis* sp. n. male genitalia. For the comparative study of the male genitalia of *Rhodnius marabaensis* sp. n. and *Rhodnius prolixus* and *Rhodnius robustus*, the descriptions by Lent and Jurberg (1969) and by Rosa et al. (2012) were used.

The following parameters of both females (15) and males (15) were measured: total length (TL); head length (HL); the length of the four antennal segments (A_1_, A_2_, A_3_, and A_4_); the three segments of the proboscis (R_1_, R_2_, and R_3_); the inner distance between the eyes (IE); the outer distance between the eyes (OE); the diameter of the eyes (DE); the maximum width of the abdomen (MWA); the maximum width of the thorax (MWT); and the length, width, and diameter of the opercular opening of the eggs (Lent and Wygodzinsky 1979, Dujardin et al. 1999, Rosa et al. 2000).

## Taxonomy

### Family Reduviidae Latreille, 1807 Subfamily Triatominae Jeannel, 1919 Genus *Rhodnius* Stål, 1859

#### Rhodnius marabaensis sp. n.

Taxon classificationAnimaliaHemipteraReduviidae

http://zoobank.org/883B9B62-9E78-4AFF-9518-021593A308A4

##### Holotype

♂. **BRAZIL: Pará**: Marabá: Reserva Ambiental Murumurú, 10°10'05.1"S, 63°24'09.1"W, 12 May 2014, N. C. B. Von Atzingen, M. B. Furtado, UNESP.

##### Paratypes.

1 ♀: same data as holotype (UNESP). 14 ♀, 14 ♂ **BRAZIL**: Pará: Jacundá/Jatobal/Marabá,N.C.B. Von Atzingen, Maraba Cultural Center Foundation – MCCF.

##### Synonym.

*Rhodnius jacundaensis* Serra, Serra and Von Atzingen (1980) [*nomen nudum*].

##### Etymology.

The name *Rhodnius marabaensis* was chosen because this species was found in the city of Marabá, Pará, Brazil.

##### Diagnosis.

The most notable morphological features that distinguish *Rhodnius marabaensis* sp. n. are the keel-shaped apex of the head, this feature is not accentuated in *Rhodnius prolixus* or *Rhodnius robustus* ; the second antennal segment of *Rhodnius marabaensis* sp. n. is 10.3 times larger than the first; in *Rhodnius prolixus*, it is 6.2 times larger, and in *Rhodnius robustus* it is 8.3 times larger. The prosternum has a longer and more clearly shaped stridulatory sulcus relative to those of *Rhodnius prolixus* and *Rhodnius robustus*. In *Rhodnius marabaensis* sp. n. the transverse carinae that border the mesosternum and the metasternum are elevated and prominent, and possess convex curvature in the central portion. In *Rhodnius prolixus*, they are less elevated and prominent, and in *Rhodnius robustus* they are interrupted in the central portion. The set of dark brown spots presents in the ventral abdomen of *Rhodnius marabaensis* sp. n. does not appear in *Rhodnius prolixus* or *Rhodnius robustus*. The ventral connective is also distinct among the three species: the black spots are smaller and, on the sixth segment, much smaller in *Rhodnius marabaensis* sp. n. The apex of the endosoma of male genitalia of *Rhodnius marabaensis* sp. n. was found to be long and straight, in *Rhodnius prolixus*, the apex is long and convex, and in *Rhodnius robustus* it is shorter, wide, and convex. The posterior surface and the ventral surface of the ninth and tenth segments of external female genitalia are distinct in the three species. *Rhodnius marabaensis* sp. n. eggs possess chorion rims, whereas those of *Rhodnius prolixus* and *Rhodnius robustus* do not (Figs 1, 3, 5, 6, 7, 10, 11, 12, 13).

##### Description.

Measurements of 15 females and 15 males of *Rhodnius marabaensis* sp. n., *Rhodnius prolixus*, and *Rhodnius robustus* are detailed in the Table 1.

**Table 1.** Mean of measurement (mm) of 15 females and males of *Rhodnius marabaensis* sp. n., *Rhodnius prolixus*, and *Rhodnius robustus*.

	Male			Female		
	*Rhodnius marabaensis*	*Rhodnius prolixus*	*Rhodnius robustus*	*Rhodnius marabaensis*	*Rhodnius prolixus*	*Rhodnius robustus*
HL	**4.90** ^a^	3.87 ^b^	3.82 ^c^	**5.32** ^a^	3.90 ^b^	4.06 ^c^
IE	0.62 ^a^	0.53 ^b^	0.64 ^a^	0.59 ^a^	0.56 ^b^	0.67 ^c^
AO	2.21 ^a^	2.05 ^b^	2.23 ^c^	3.04 ^a^	2.26 ^b^	2.38 ^c^
PO	0.98 ^a^	0.92 ^b^	0.72 ^c^	1.06 ^a^	0.77 ^b^	0.78 ^c^
DE	1.72 ^a^	1.94 ^b^	1.00 ^c^	1.91 ^a^	1.68 ^b^	1.64 ^c^
R1	0.97 ^a^	0.55 ^b^	0.91 ^c^	0.93 ^a^	0.57 ^b^	0.97 ^c^
R2	**3.87** ^a^	3.25 ^b^	3.02 ^c^	**3.77** ^a^	3.32 ^b^	3.30 ^c^
R3	0.87 ^a^	0.33 ^b^	0.92 ^c^	0.77 ^a^	0.39 ^b^	0.96 ^c^
TL	**20.41** ^a^	19.98 ^b^	20.20 ^c^	**22.35** ^a^	20.98 ^b^	21.28 ^c^
MWT	4.25 ^a^	4.62 ^b^	4.06 ^c^	4.88 ^a^	4.82 ^b^	4.12 ^c^
MWA	**6.22** ^a^	5.93 ^b^	6.03 ^c^	**6.92** ^a^	6.75 ^b^	6.56 ^c^
A1	0.48 ^a^	0.38 ^b^	0.37 ^c^	0.45 ^a^	0.37 ^b^	0.38 ^c^
A2	**4.72** ^a^	3.04 ^b^	3.28 ^c^	**4.47** ^a^	2.88 ^b^	3.18 ^c^
A3	2.68 ^a^	2.25 ^b^	2.32 ^c^	3.05 ^a^	1.94 ^b^	2.41 ^c^
A4	1.05 ^a^	0.98 ^b^	1.54 ^c^	1.15 ^a^	0.94 ^b^	1.64 ^c^

HL : head length ; IE : inner distance between eyes ; AO : anteocular distance ; PO : postocular distance (excluding neck); DE : diameter of the eye ; R1, R2 and R3: lengths of first, second and third rostral; TL : total length of the triatomine ; MWT : maximum width of the thorax ; MWA : maximum width of the abdomen ; A1, A2, A3 and A4: 1^st^, 2^nd^, 3^rd^, and 4^th^ antennal segments, respectively; a,b,c: Lower case letters indicate significant differences between specimens with Tukey's test: p < 0,05. Values in bold indicate the main findings.

Head with apex (central longitudinal dorsal portion), which is elevated, straw yellow, and keel shaped. This keel-shaped section presents the same shape from the beginning of the clypeus to the posterior portion of the ocelli; thus, the border of the clypeus is visible around/from the gena and the jugum (1+1), which are located laterally. However, the gena begin before the beginning of the clypeus. Thus, the gena go toward the anteclypeus which are rounded. The species presents crystalline ocelli and eyes with black and yellow ommatidia. The first and second segments of the antennae are yellow, whereas the posterior two thirds of the third segment are white, and the fourth segment is completely white. The species presents a second antennal segment that is significantly larger than the others (10.3 times larger than the first; 1.65 times larger than the third, and 4.3 times larger than the fourth) (Table 1).

At the juncture between the neck and the thorax, there is a ring that is anteriorly black and posteriorly yellow; the anterolateral angles (1+1) are yellow. The dorsal thorax (pronotum) is shaped like a trapezoid and surrounded by a yellow carina. There are two yellow submedian carinae running lengthwise around the pronotum, from the anterior portion to the posterior one. The submedian carina border three anterior lobes, each of which has a set of black spots, and three posterior lobes with two parallel black stripes on each that are connected to the set of black spots on the anterior lobes. The triangular scutellum is marked laterally and is very clear because of its black color. The internal portion is also triangular and yellow, and it is bordered by thick and obvious carina. When the wings are removed, the posterior portion (tip) of the scutellum covers 2/3 of the I urotergite process (Figs 4, 5).

From the ventral surface of the thorax, a prosternum with deep, well-defined stridulatory black sulcus is visible; in the posterior portion, it takes on a funnel shape and ends as a tip between the anterior pair of legs (Fig. 5). The mesosternum has two elevated black areas that are separated by a yellow depression. The border between the mesosternum and the metasternum is formed by a set of three elevated and prominent carinae. The two lateral carinae are black, and the central carina is yellow. These three carinae are curved backward. The central carina, which is elevated and prominent, possesses a semicircular depression in the central portion at the border with the metasternum. The metasternum is slightly rectangular in shape. The central portion is black and outlined by two yellow carinae (Fig. 6).

The legs have an overall yellowish tone. The coxae have yellow and black spots; the trochanters are yellow and do not have spots; the femurs are yellow with black spots running lengthwise; the tibias are yellow except for the posterior sixth segment, which continues the black pattern of the tarsi (Fig. 2).

The ventral surface of the abdomen is predominantly yellow, with three sets of black stripes: one on the central longitudinal portion and (2+2) on the side portions above the connectives (Fig. 2). The first abdominal segment has a longitudinal black spot between the two larger yellow spots. The second, third, fourth, and fifth ventral abdominal segments possess (1+1) curved sets of dark brown spots. These spots begin at the anterior dividing line and extend diagonally along the central portion of the segments (Fig. 2). The dorsal connective includes yellow and black spots that cover half of each segment. They are wide in the anterior portion and become thinner in the inner posterior portion. The black spot of the connective of the sixth segment is smaller than those of the fifth, fourth, third, and second segments (Fig. 2). The first tergite, which is visible when the wings are removed, is essentially formed by two parts. The anterior portion has a striated cuticula that contrasts with the surrounding smooth cuticula and which is triangular in shape on its upper level. It possesses a clearly defined transverse sulcus. The second portion is posterior to the first and is at a lower level. It consists of a set of transverse and straight fringes (Fig. 5).

When the male genitalia is seen from the dorsal surface, it is clear that the phallus (P) is formed by an endosoma (En), by the median extension of the basal plate (EPlb), and by the basal piece itself (Plb). When seen from the ventral surface, the phallus (P) is formed by the conjunctiva (Cj), the phallosoma (ph), the phallosoma process (PrPh), the median extension of the basal piece (EPlb), the gonopore process (PrG), and the basal piece (Plb). When seen from the lateral surface, the phallus is formed by the conjunctiva (Cj), the endosoma (En), the median extension of the basal piece (EPlb), the basal piece itself (Plb), the phallosoma (Ph), and the phallosoma process (PrPh) (Fig. 8).

The dorsal surface of the external female genitalia was examined using scanning electron microscopy (SEM), which showed that the seventh segment is separated from the eighth segment by a slightly irregular line and forms (1+1) triangular tips at the border between the connective and the eighth segment. The eighth segment is trapezoid shaped. The ninth segment appears as a protrusion. The tenth segment appears as a small curve in the central portion where it delimits with the eighth segment (Fig. 9)

From the posterior surface, (1+1) appendages can be seen on the border between the eighth and ninth segments. The tenth segment is semicircular in shape with a pronounced central slit in the shape of an upside-down V and with (1+1) protrusions at the posterior edge of the gonocoxite 8. Display is also a (1+1) gonocoxite 8 and a (1+1) gonapophysis 8 (Fig. 10).

From the ventral surface, the lateral portions of the line that divides the seventh segment and the gonocoxites 8 and the gonapophysis 8 are curved, and the line then forms small (1+1) ascending curves and a slight depression in the central portion. The ninth segment forms (1+1) lateral flaps at the border with the tenth segment and presents transverse slits at the sub-intermediate position (1+1). The transverse slits then form into two triangles, whose tips are separated in the central portion. The tenth segment is the outer edge of the external female genitalia and is presented as a narrow curved and convex band (Fig. 11).

The egg shells measure 1.59 mm in length and 0.71 mm in width. They present prominent collar and chorial rim (Fig. 12 and Table 2). The exochorion cells are clearly demarcated, with internal granulations organized into a circle. The follicular tubes of each exochorion cell do not differ in diameter (Fig. 13).

**Table 2.** Mean of measurements (mm) of 13 eggs of *Rhodnius marabaensis* sp. n., *Rhodnius prolixus*, and *Rhodnius robustus*.

Measurement	*Rhodnius marabaensis*	*Rhodnius prolixus*	*Rhodnius robustus*
L (mm)	1.54 ± 0.04 ^a^	1.73 ± 0.02 ^b^	1.61 ± 0.04 ^c^
W (mm)	0.87 ± 0.01 ^a^	0.71 ± 0.06 ^b^	0.93 ± 0.01 ^c^
Oo (mm)	0.49 ± 0.01 ^a^	0.67 ± 0.01 ^b^	0.73 ± 0.01 ^c^

L : length (40×); W : width (40×); Oo : opening of operculum (80×); a,b,c: Lower case letters indicate significant differences between specimens with Tukey's test: p < 0,05.

The molecular study shown the same haplotype for the find sequences of the cyt-b (693 bp) and the evaluation by BLAST have shown that *Rhodnius marabaensis* sp. n. is closely related to *Rhodnius robustus* and *Rhodnius prolixus* (until 99% and 94% of identity, respectively).

## Discussion

In epidemiological terms (i.e., considering the role of the species as vectors of *Trypanosoma cruzi*), the three main Triatominae genera are *Panstrongylus*, *Rhodnius*, and *Triatoma*. Distinguishing among these three genera is not difficult because they can be characterized macroscopically based on the format of the head and the position of the antenniferous tubercle, as described by Pinto (1931). However, distinguishing among *Rhodnius* species requires a more detailed examination through optical microscopy. The difficulty in identifying *Rhodnius* species was first noted by Neiva and Pinto (1923). At the time, there were five known species: *Rhodnius nasutus*, *Rhodnius prolixus*, *Rhodnius pictipes*, *Rhodnius brethesi*, and *Rhodnius domesticus* (Lent and Wygodzinsky 1979). Including this description of *Rhodnius marabaensis* sp. n., there are currently 20 species (Rosa et al 2012, Abad Franch 2013); therefore, specific distinction is even more difficult and requires more characteristics to be considered. As a result, the characterization of *Rhodnius marabaensis* sp. n. is discussed comparatively using *Rhodnius prolixus* and *Rhodnius robustus*. *Rhodnius marabaensis* is closely related to *Rhodnius robustus* and *Rhodnius prolixus* (until 99% and 94% of identity, respectively). In view of this and of the fact that all three species occur in Northern Brazil (Galvão 2014), *Rhodnius robustus* and *Rhodnius prolixus* were morphologically examined and compared in more detail (Table 3).

**Table 3.** Triatominae species found in the Amazon region.

Species	Descriptors	Distribution	References
*Rhodnius amazonicus*	Almeida, Santos & Sposina, 1973	Brazil (Amazonas), French Guiana (Cacao, Saul)	Galvão 2014
*Rhodnius barretti*	Abad-Franch, Palomeque & Monteiro, 2013	Colombia (Puerto de Assís), Ecuador (Lagro Ágrio)	Abad-Franch et al. 2013
*Rhodnius brethesi*	Matta, 1919	Brazil (Amazonas), Venezuela (Aragua)	Galvão 2014
*Rhodnius colombiensis*	Mejia, Galvão, Jurberg, 2009	Colombia (Tolima)	Guhl et al. 2007
*Rhodnius dalessandroi*	Carcavallo & Barreto, 1976	Colombia (Meta)	Guhl et al. 2007
*Rhodnius ecuadoriensis*	Lent & Leon, 1958	Ecuador (Manabi, Eujias, Loja), Peru (Amazonas, Tumbis, Piura, Cojamarca, La libertad, Lambayeque e San Martin)	Galvão et al. 2003
*Rhodnius neivai*	Lent, 1953	Colombia (Cesar), Venezuela (Lara, Falcón, Zulia)	Galvão et al. 2003
*Rhodnius milesi*	Carcavallo et al., 2001	Brazil (Pará)	Galvão 2014
*Rhodnius montenegrensis*	Rosa et al., 2012	Brazil (Rondônia, Acre)	Galvão 2014
*Rhodnius pallescens*	Barber, 1932	Colombia (Bolívar, Sucre)	Arboleda et al. 2009
*Rhodnius paraensis*	Sherlock, Guitton & Miles, 1977	Brazil (Amazonas,Pará), French Guiana	Galvão 2014
*Rhodnius pictipes*	Stal, 1872	Brazil, French Guiana, Colombia, Peru, Suriname, Venezuela	Galvão 2014
*Rhodnius prolixus*	Stal, 1859	Brazil, Bolivia, Colombia, Guatemala	Galvão et al. 2003
*Rhodnius robustus*	Larousse, 1927	Brazil, Bolivia, Colombia, Venezuela, French Guiana	Galvão 2014
*Rhodnius stali*	Lent, Jurberg & Galvão, 1993	Brazil (Mato Grosso do Sul, Acre), Bolivia (Santa Cruz, La Paz)	Matias et al. 2003

Source: Galvão 2014; Galvão et al. 2003; Matias et al. 2003.

*Rhodnius marabaensis* sp. n. is predominantly yellow, whereas *Rhodnius prolixus* is black and *Rhodnius robustus* is brown. The legs of the three species are also consistent in these color schemes (Fig. 2). Another very clear characteristic of *Rhodnius marabaensis* sp. n. is that it is larger than *Rhodnius prolixus* and *Rhodnius robustus* (Figs 3–5 and Table 1).

The head of *Rhodnius marabaensis* sp. n. has four very clear characteristics that help to distinguish it from the others:

1. It possesses a keel-shaped longitudinal dorsal portion (apex) running from the clypeus to the ocelli. This feature is not accentuated in *Rhodnius prolixus* or *Rhodnius robustus* (Fig. 3);
2. It possesses a rounded anteclypeus. In *Rhodnius prolixus* and *Rhodnius robustus*, the anteclypeus is flat (Fig. 3);
3. It possesses an indistinguishable clypeus. In *Rhodnius prolixus*, it is narrow in the anterior portion and wide in the posterior one. In *Rhodnius robustus* it is wide in the anterior portion, narrow in the medial portion, and wide in the posterior one (Fig. 3);
4. The second segment of the antenna is significantly larger than the other three, with the following length ratio among the four antennal segments: 2nd > 3rd > 4th > 1st. This relative length pattern of the four antennal segments is the same as the pattern observed by Rosa et al. (2010) in *Rhodnius neglectus* and *Rhodnius prolixus* adults; however, the differences in size between the largest and the smallest segments are distinct: the second antennal segment of *Rhodnius marabaensis* sp. n. is 10.3 times larger than the first; in *Rhodnius prolixus*, it is 6.2 times larger, and in *Rhodnius robustus* it is 8.3 times larger (Table 1).

For the description of *Rhodnius marabaensis* sp. n. five features of the thorax allow for its distinction:

1. The scutellum is larger and includes two prominent internal lateral carinae. It is therefore distinct from *Rhodnius prolixus* and *Rhodnius robustus*, which present smaller scutellum and whose carinae are not pronounced (Fig. 4);
2. The first urotergite has a pronounced transverse groove and inferior fringe consisting of long and straight filaments. In *Rhodnius prolixus*, the transverse groove does not appear and the fringe possesses short and irregular filaments. On the other hand, in *Rhodnius robustus* the transverse groove is not accentuated, and the fringe possesses short and straight filaments, as shown in (Fig. 5).
3. It possesses a longer and more clearly shaped stridulatory sulcus relative to those of *Rhodnius prolixus* and *Rhodnius robustus* (Fig. 5). This specific differentiation among these three *Rhodnius* species adds to the observations by Lent and Wygodzinsky (1979), who verified that the stridulatory sulcus in six species from different genera presented characteristics that allowed for species identification;
4. The transverse carinae that border the mesosternum and the metasternum differ among the three species. In *Rhodnius marabaensis* sp. n., they are elevated and prominent, and possess convex curvature in the central portion. In *Rhodnius prolixus*, they are less elevated and prominent, and in *Rhodnius robustus* they are interrupted in the central portion (Fig. 6);
5. The metasternum is slightly rectangular in shape in *Rhodnius marabaensis* sp. n., whereas in *Rhodnius prolixus* and *Rhodnius robustus* they are slightly triangular (Fig. 6).

From the view of the ventral surface, the abdomen of *Rhodnius marabaensis* sp. n. is distinct because of its yellow colouring; this area is brown in *Rhodnius prolixus* and black in *Rhodnius robustus* (Fig. 11). The set of dark brown spots does not appear in *Rhodnius prolixus* or *Rhodnius robustus*, and the ventral connective is also distinct among the three species: the black spots are smaller and, on the sixth segment, much smaller in *Rhodnius marabaensis* sp. n. (Fig. 2).

When the male genitalia was examined from the dorsal surface, the apex of the endosoma of *Rhodnius marabaensis* sp. n. was found to be long and straight; in *Rhodnius prolixus*, the apex is long and convex, and in *Rhodnius robustus* it is shorter, wide, and convex (Lent and Jurberg 1969) (Fig. 7). The analysis also revealed that the two final portions of the basal piece are turned to the side in *Rhodnius marabaensis* sp. n., whereas in *Rhodnius robustus* they face the posterior region. From the ventral surface, the phallosoma of *Rhodnius marabaensis* sp. n. is rounded, whereas in *Rhodnius prolixus* the anterior portion is convex and in *Rhodnius robustus* it is square (Fig. 7). The pygophore of *Rhodnius marabaensis* sp. n., *Rhodnius prolixus*, and *Rhodnius robustus* are all in the shape of an isosceles triangle. However, the sides of the pygophores are straight in *Rhodnius robustus* and curved in *Rhodnius prolixus* and *Rhodnius marabaensis* sp. n. The pygophore of *Rhodnius marabaensis* sp. n. is also larger at the tip, thicker, and circular (Fig. 8).

The external female genitalia was also considered. From the dorsal surface, *Rhodnius marabaensis* sp. n. and *Rhodnius robustus* show no differences; however, the format of the ninth segment of the dorsal surface in *Rhodnius prolixus* presents different characteristics (Fig. 9). The posterior surface and the ventral surface of the ninth and tenth segments are distinct in the three species (Fig. 10). These characteristics are also distinct from 12 other *Rhodnius* species, as described by Rosa et al. (2014).

*Rhodnius marabaensis* sp. n. eggs possess chorion rims, whereas those of *Rhodnius prolixus* and *Rhodnius robustus* do not. The diameter of the follicular tubes of the exochorion cells in *Rhodnius prolixus* are larger than those of *Rhodnius marabaensis* sp. n. and *Rhodnius robustus*; however, they are regular in *Rhodnius marabaensis* sp. n. and varied in *Rhodnius robustus* (Figs 12, 13 and Table 2).

The data obtained corroborate the status of *Rhodnius marabaensis* sp. n. as a new species and indicates that the use of morphology for the description of Triatominae species offers phenotypic information (morphological and morphometric) to define the status of species. It is also important to note that molecular analysis generates data that can help phylogenetic relationships and taxonomic studies of Triatominae (Abad-Franch and Monteiro 2005, Justi et al. 2014). BLAST analysis of cyt-b sequence shows *Rhodnius marabaensis* sp. n. as closely related to *Rhodnius robustus* and *Rhodnius prolixus*, so this new species must be included in the *Rhodnius prolixus* complex (Carcavallo et al. 2000). Complementary approaches using molecular data must be encouraged to establish the phylogenetic placement of this new species based on the evaluation of other gene fragments and a more robust assessment.

## Supplementary Material

XML Treatment for Rhodnius marabaensis

## References

[B1] Abad-Franch F Monteiro FA (2005) Molecular research and the control of Chagas disease vectors. Annals of the Brazilian Academy of Sciences 77: 437–454. doi: 10.1590/S0001-37652005000300007 10.1590/s0001-37652005000300007 16127551

[B2] Abad-Franch F Pavan MG Jaramillo O Palomeque FS Dale C Chaverra D Monteiro FA (2013) *Rhodnius barretti*, a new species of Triatominae (Hemiptera: Reduviidae) from western Amazonia. Memórias Instituto Oswaldo Cruz 108: 92–99. doi: 10.1590/0074-0276130434 10.1590/0074-0276130434 PMC4109185 24473808

[B3] Alevi KCC Ravazi A Mendonça VJ Rosa JA Azeredo-Oliveira MTV (2015) Karyotype of *Rhodnius montenegrensis* (Hemiptera, Triatominae). Genetics and Molecular Research 12: 222–226. doi: 10.4238/2015.January.16.5 10.4238/2015.January.16.5 25729953

[B4] Almeida FB Santos EI Sposina G (1973) Triatomíneos da Amazonia III. Acta Amazonica 3: 43–66.

[B5] Bérenger JM Pluot-Sigwalt D (2002) *Rhodnius amazonicus* Almeida, Santos & Sposina, 1973, bona species, close to *R. pictipes* Stål, 1872 (Heteroptera: Reduviidae: Triatominae). Memórias do Instituto Oswaldo Cruz 97: 73–77. doi: 10.1590/S0074-02762002000100011 11992151 10.1590/s0074-02762002000100011

[B6] Carcavallo RU Galvão C Lent H (1998) *Triatoma jurbergi* sp.n. do Norte do Estado do Mato Grosso, Brasil (Hemiptera, Reduviidae, Triatominae) com uma Atualização das Sinonímias e outros Táxons. Memórias do Instituto Oswaldo Cruz 93: 459–464. doi: 10.1590/S0074-02761998000400007 9711334 10.1590/s0074-02761998000400007

[B7] Carcavallo RU Jurberg J Lent H Noireau F Galvão C (2000) Phylogeny of the Triatominae (Hemiptera: Reduviidae). Proposals for taxonomic arrangements. Entomología y Vectores 7: 1–99.

[B8] Dujardin J Steinden M Chavez T Machane M Schofield C (1999) Changes in the Sexual Dimorphism of Triatominae in the Transition From Natural to Artificial Habitats. Memórias Instituto Oswaldo Cruz 94: 565–569. doi: 10.1590/S0074-02761999000400024 10.1590/s0074-02761999000400024 10446020

[B9] Galvão C Carcavallo R Rocha DS Juberg JA (2003) Checklist of the current valid species of the subfamily Triatominae Jeannel, 1919 (Hemiptera, Reduviidae) and their geographical distribuition, with nomenclatural and taxonomic note. Zootaxa 202: 1–36. doi: 10.11646/zootaxa.202.1.1

[B10] Galvão C (2015) Vetores da doença de Chagas no Brasil. Sociedade Brasileira de Zoologia, Curitiba, 289 pp.

[B11] Jurberg J Rocha DS Galvão C (2009) *Rhodnius zeledoni* sp. nov. afim de *Rhodnius paraensis* Sherlock, Guitton e Milles, 1977 (Hemíptera, Reduviidae, Triatominae). Biota Neotropica 9: 123–128. doi: 10.1590/S1676-06032009000100014

[B12] Justi AS Russo CAM Mallet JRS Obara MT Galvão C (2014) Molecular phylogeny of Triatomini (Hemiptera: Reduviidae: Triatominae). Parasites & Vectors 7: . doi: 10.1186/1756-3305-7-149 10.1186/1756-3305-7-149 PMC4021723 24685273

[B13] Lent H Juberg J (1969) O gênero *Rhodnius* Stål, 1859 com um estudo sobre a genitália das espécies (Hemiptera, Reduviidae, Triatominae). Brazilian Journal of Biology 29: 487–560.

[B14] Lent H Wygodzysnky P (1979) Revision of the Triatominae (Hemiptera - Reduviidae) and their significance as vectors of Chagas’ disease. Bulletin of the American Museum of Natural History 163: 125–520.

[B15] Lent H Juberg J Galvão C (1993) *Rhodnius stali* n. sp., Afim de *Rhodnius pictipes*, 1872 (Hemíptera, reduviidae, triatominae). Memórias do Instituto Oswaldo Cruz 88: 605–614. doi: 10.1590/S0074-02761993000400019

[B16] Meija JM Galvão C Juberg J (1999) *Rhodnius colombiensis* sp. n. da Colômbia, com quadros comparativos entre estruturas fálicas do gênero *Rhodnius* Stål, 1859 (Hemíptera, Reduviidae, Triatominae). Entomología y Vectores 6: 601–617.

[B17] Mendonça VJ Chaboli AKC Pinotti H Gurgel-Gonçalves R Pita S Guera AL Panzera F Araújo RF Vilela de Azeredo-Oliveira MT Rosa JA (2016) Revalidation of *Triatoma bahiensis* Sherlock & Serafim, 1967 (Hemiptera: Reduviidae) and phylogeny of the *T. brasiliensis* species complex. Zootaxa 4107: 239–254. doi: 10.11646/zootaxa.4107.2.6 27394816 10.11646/zootaxa.4107.2.6

[B18] Meneguetti DUO Castro GVS Castro MALR Souza JLS Oliveira J Rosa JA Aranha Camargo LMA (2016) First report of *Rhodnius stali* (Hemiptera, Reduviidae, Triatominae) in the State of Acre and in the Brazilian Amazon. Journal of the Brazilian Society of Tropical Medicine 49: 365–368. doi: 10.1590/0037-8682-0066-2016 27384836 10.1590/0037-8682-0066-2016

[B19] Monteiro FA Barrett TV Fitzpatrick S Cordon-Rosales C Feliciangeli D Beard CB (2003) Molecular Phylogeography of the Amazonian Chagas disease vectors *Rhodnius prolixus* and *Rhodnius robustus*. Molecular Ecology 12: 997–1006. doi: 10.1046/j.1365-294X.2003.01802.x 12753218 10.1046/j.1365-294x.2003.01802.x

[B20] Neiva A Pinto C (1923) Estado actual dos conhecimentos sobre o gênero *Rhodnius* Stål, com a descrição de uma nova espécie. Brasil-Médico 37: 20–24.

[B21] Pinto C (1931) Valor do rostro e antenas na caracterização dos gêneros de triatomideos. (Hemiptera, Reduviidae). Boletim biológico de São Paulo 19: 45–136.

[B22] Rosa JA Freitas SC Malara FF Rocha CS (2010) Morphometry and morphology of the antennae of *Panstrongylus megistus* Burmeister, *Rhodnius neglectus* Lent, *Rhodnius prolixus* Stål and *Triatoma vitticeps* Stål (Hemiptera: Reduviidae). Neotropical Entomology 39: 214–220. doi: 10.1590/S1519-566X2010000200011 20498958 10.1590/s1519-566x2010000200011

[B23] Rosa JA Barata JMS Santos JLF Cilense M (2000) Morfologia de ovos de *Triatoma circummaculata* e *Triatoma rubrovaria* (Hemiptera, Reduviidae) Egg morphology of *Triatoma circummaculata* and *Triatoma rubrovaria* (Hemiptera, Reduviidae). Journal of Public Health 34: 538–542. 11105119 10.1590/s0034-89102000000500015

[B24] Rosa JA Mendonça VJ Rocha CS Gardim S Cilense M (2010) Characterization of the external female genitalia of six species of Triatominae (Hemiptera: Reduviidae) by scanning electron microscopy. Memórias do Instituto Oswaldo Cruz 105: 286–292. doi: 10.1590/S0074-02762010000300007 20512241 10.1590/s0074-02762010000300007

[B25] Rosa JA Rocha CS Gardim S Pinto MC Mendonça VJ Filho JCRF Carvalho EOC Camargo LMA Oliveira JO Nascimento JD Cilense M Almeida CD (2012) Description of *Rhodnius montenegresis* n. sp. (Hemiptera: reduviidae: Triatominae) from the state of Rondônia, Brazil. Zootaxa 3478: 62–76.

[B26] Rosa JA Mendonça VJ Gardim S Blanco DC Oliveira J Nascimento JD Pinotti H Pinto MC Cilense M Galvão C Barata JMS (2014) Study of the external female genitalia of 14 *Rhodnius* species (Hemiptera, Reduviidae, Triatominae) using scanning electron microscopy. Parasite & Vectors 7: . doi: 10.1186/1756-3305-7-17 10.1186/1756-3305-7-17 PMC3896706 24405517

[B27] Serra OP Serra RG Atzigen NCBV (1980) Nova espécie do gênero *Rhodnius* da Amazônia, Estado do Pará, Brasil. Anais do V Congresso Brasileiro de Parasitologia, Rio de Janeiro, 120 pp.

[B28] Valente VC Valente S Carcavallo RU Rocha DS Galvão C Juberg J (2001) Considerações sobre uma nova espécie do gênero *Rhodnius* Stål, do estado do Pará, Brasil (Hemíptera, Reduviidae, Triatominae). Entomología y Vectores 8: 65–80.

